# Type of Work and Preoperative Ability to Perform Work Affect Return to Usual Work Following Proximal Interphalangeal Joint Arthroplasty for Osteoarthritis

**DOI:** 10.1177/15589447221141485

**Published:** 2022-12-20

**Authors:** Bo J. W. Notermans, Joris S. Teunissen, Ruud W. Selles, Luitzen H. L. de Boer, the Hand Wrist Study Group, Brigitte E. P. A. van der Heijden

**Affiliations:** 1 Radboudumc, Nijmegen, The Netherlands; 2 Erasmus MC, Rotterdam, The Netherlands; 3 Xpert Clinics, Eindhoven, The Netherlands; 4 Jeroen Bosch Ziekenhuis, ‘s-Hertogenbosch, The Netherlands

**Keywords:** osteoarthritis, return to work, PIP, arthroplasty, implant

## Abstract

**Background:**

The time until return to work (RTW) and possible factors affecting this time after proximal interphalangeal (PIP) joint arthroplasty are unknown. Therefore, we aim to evaluate the RTW after PIP joint arthroplasty for osteoarthritis and assess factors affecting the time until return to their usual work.

**Methods:**

We used prospectively gathered data from 74 patients undergoing PIP joint arthroplasty with daily hand surgery practice routine outcome collection. Standardized RTW questionnaires were completed at 6 weeks and 3, 6, and 12 months after surgery. Return to work was defined as the first time a patient reported returning to work and performing the original work for a minimum of 50% of the original hours a week, as stated in the patient’s contract. Second, we evaluated baseline factors affecting the time until RTW.

**Results:**

The probability of RTW within 12 months after surgery was 88%. The median time until RTW was 8 weeks (interquartile range: 4-10). Physical occupational intensity (hazard ratio [HR]: 0.36, *P* = .001) and the baseline Michigan Hand Outcomes Questionnaire work scores (HR: 1.02, *P* = .005) were independently associated with RTW.

**Conclusion:**

In conclusion, patients returned to work after a median of 8 weeks following PIP arthroplasty. Patients with medium or heavy physical occupations returned to work later than patients with light physical occupations. Better patient-reported work outcomes at baseline also led to an earlier RTW. This information can be valuable for providing adequate information during the preoperative consultation.

## Introduction

Proximal interphalangeal (PIP) joint arthroplasty is a motion-sparing surgical treatment for patients with symptomatic PIP joint osteoarthritis. In a recent study, we reported a clinically meaningful improvement of pain among 60% of patients with preservation of PIP joint range of motion and high satisfaction rates during the first year after PIP joint arthroplasty for osteoarthritis.^1^

Most research has focused on survival and revision rates of PIP implant surgery, complications, and range of motion.^2-17^ There is a knowledge gap concerning return to work (RTW) after PIP joint arthroplasty. This knowledge is vital to create adequate patient expectations on rehabilitation.

Return to work studies have been performed for other hand disorders, and injuries and several prognostic factors, such as sex, type of work, and the amount of postoperative pain, for RTW were found.^18-21^ For instance, among patients undergoing surgical treatment for trapeziometacarpal osteoarthritis, the physical occupational intensity, surgery on the dominant hand, and better Michigan Hand Outcomes Questionnaire (MHQ) work score and hand function score of the opposite hand preoperatively led to a shorter time until patients returned to work.^22^ Which factors are of importance for patients’ RTW following PIP joint arthroplasty for osteoarthritis is unknown. Therefore, this study describes the RTW after PIP joint arthroplasty and identifies factors that influence the time until RTW. We are specifically interested in the baseline factors, so that we can estimate time until RTW preoperatively.

## Patients and Methods

### Study Design and Setting

Data of patients who underwent PIP arthroplasty were gathered prospectively between January 1, 2009, and October 1, 2019, in daily hand surgery practice, reported following the Strengthening the Reporting of Observational Studies statement.^23^ When visiting our institution, patients were invited to participate in a routine outcome measurement system and provided written consent to use their data for clinical research. Patients received questionnaires at baseline and 6 weeks, 3, 6, and 12 months after surgery using the Generic Medical Survey Tracker (GemsTracker) electronic data capture tool.^24^ GemsTracker is a secure Web-based application for distributing questionnaires and forms during clinical research and quality registrations; details have been published earlier.^25^

Surgery was performed by 22 surgeons, who were all hand fellowship–trained, and most were Federation of European Societies for Surgery of the Hand-certified. Five surgeons operated on most patients (n = 48 of the total of 74 included patients). Their experience ranged from level 3 to level 5 according to the classification by Jin Bo Tang and Giddins.^26^ The local medical research ethical committee approved this study.

### Patients

We included patients who underwent a PIP joint arthroplasty for either primary degenerative or post-traumatic osteoarthritis (after intra-articular PIP fractures) and had paid employment before surgery. Patients were considered for surgery if they had radiological signs of PIP joint osteoarthritis (Kellgren-Lawrence classification ≥grade 2) in combination with pain, despite nonsurgical treatment for at least 3 months.^27^ Besides pain, stiffness and deformity could be indications for surgery. We excluded patients with inflammatory arthritis because it concerns an autoimmune disease, for which treatment also includes medication involving multiple joints in multiple body parts, which may influence the capability of performing work in general. Besides, we excluded patients who did not complete the RTW questionnaires at least once.

### Surgical Procedure

Surgery is performed under local anesthesia or with a regional local anesthetic block (axillary or supraclavicular) in a bloodless field (with a tourniquet). In all patients, a dorsal longitudinal skin incision is made over the PIP joint, and the central tendon is split longitudinally (n = 67) or according to Chamay (n = 7) to expose the joint.^28^ Despite the difference in joint exposure, the same surgical procedure was performed. After preparing the joint, a trial prosthesis is introduced. Function and stability is tested, and a permanent implant is chosen: silicone (NeuFlex DePuy Orthopaedics, Warsaw, Indiana) or surface replacement (SR; Avanta, Avanta Orthopaedics, San Diego). Position and alignment are checked by radiography in 2 positions (posteroanterior and lateral). The extensor tendon is repaired with absorbable sutures. The skin is closed using nonabsorbable sutures, and an extension splint is applied.

### Postoperative Rehabilitation

Given the observational nature of this study, the surgical procedure and postoperative rehabilitation are not as standardized as in randomized controlled trials. However, standardized treatment protocols were used (Supplemental Table 1). Patients return to the hand therapist 3 to 5 days postoperatively. The cast is removed, and an extension splint at 0° is made. In the case of hyperextension, the splint is adjusted with a 10° to 30° extension block, depending on the severity. Physical therapy is initiated, starting with active exercise therapy without resistance during the first 8 weeks. The daytime use of the extension splint is phased out after 7 to 8 weeks, but it is continued at night. After 8 weeks, exercise with resistance is built up slowly based on patients’ symptoms. A follow-up visit with the surgeon is scheduled 3 months after surgery, and a radiograph is obtained on indication. After 3 months, the use of the splint at night is reduced. Therapy continues, if needed, until 6 to 12 months after surgery.

### Data Collection

Patient and surgery characteristics recorded as part of routine outcome measurements before initiating treatment are age, sex, hand dominance, work status, dominance, and the duration of symptoms. The MHQ was used to assess patient-reported outcomes at intake.^29^ A medical chart review was performed to collect additional patient and surgery characteristics, such as diagnosis, type of implant, and accompanying hand conditions, such as polyarthritis and stenosing tendovaginitis.

Hand therapists recorded patients’ physical intensity of work, which was divided into 3 categories: light physical work (eg, an office job), moderate physical work (ie, working in a shop), and heavy physical work (eg, working at a construction site). Patients with paid employment were asked to complete the online RTW questionnaire at 6 weeks and 3, 6, and 12 months after surgery.

### Return to Work Questionnaire

The first question of the online questionnaire—whether the patient has returned to work—must be answered with yes to continue with the following 5 questions, which include questions regarding the number of work hours before and after treatment, the duration of sick leave, and whether adjustments had to be made or temporary assignment to substitutional tasks was performed.

Return to work was defined as the first time patients returned to their original work performing a minimum of 50% of the original hours a week, as stated in the patient’s contract. Thus, this excluded performing adjusted work as a criterion of RTW. We chose 50% of RTW as our primary outcome because Dutch labor laws require patients to perform less than 50% of their original work to be allowed for any form of compensation. This definition aligns with previous studies on RTW.^20-22^

### Statistical Methods

Medians and interquartile ranges are reported for non-normal distributed data, and means with 95% confidence intervals are reported for normally distributed data. Normality was assessed using histograms and Q-Q plots. We had a substantial number of nonresponding patients due to collecting data during daily clinical practice using Web-based questionnaires. Therefore, we performed a nonresponder analysis to test whether missingness was dependent on any recorded feature. Patients who completed the RTW questionnaire at least once were responders, whereas a nonresponder was defined as a patient who did not complete any RTW questionnaire. We used the χ^2^ test to compare categorical data, *t* tests for normal distributed numerical data, and Wilcoxon signed-rank tests for non-normal distributed numerical data (Supplemental Table 2).

We calculated the median time until RTW using the Kaplan-Meier method and plotted inverted survival curves. Patients who reached retirement during follow-up or did not complete the questionnaires at later time points were still included in this study for the period that they provided data, after which they were censored (marked with a “+” in the Kaplan-Meier plots), thus dealing with loss to follow-up and minimizing bias.^30^

We selected age, sex, occupational intensity, dominance, the duration of symptoms (in months), and MHQ scores at baseline as possible influencing factors of the RTW based on previously published literature.^20-22^ Besides, the authors agreed upon adding hand comorbidity, diagnosis, and implant type as possible influencing factors specifically for patients following PIP joint arthroplasty. We evaluated the median times until RTW of all factor subgroups, and in the case of continuous variables, we categorized based on the median value. We analyzed continuous baseline variables that could affect the RTW using univariable Cox proportional hazards models. A hazard ratio (HR) <1 was interpreted as a decreased probability of returning to work, whereas HR > 1 increased the probability of returning to work. The proportional hazards assumption was tested using the Schoenfeld residuals. All variables with a *P* value smaller than .10 in univariable analysis were included in the multivariable Cox proportional hazards model to assess independently associated factors with RTW. We made sure not to exceed the advised minimum of 10 events per included predictor variable.^31,32^ Significant dichotomous variables in the multivariable model were plotted univariably using the Kaplan-Meier method, and a log-rank test was performed to compare survival times between groups.

A *P* value smaller than .05 was considered statistically significant for all tests. All analyses were performed in software package R, version 3.6.1.

## Results

Within the study period, 239 patients underwent a PIP joint arthroplasty. We excluded 133 patients without paid occupation before surgery, so 106 patients could complete the RTW questionnaire. We excluded 4 patients diagnosed with inflammatory arthritis and 28 patients who did not complete the RTW questionnaire at least once. Therefore, we could include 74 patients for analysis. Seventy percent of patients were woman, and the mean age was 57 years (SD: 8.4). Fifty-five percent of patients had hand comorbidities, such as polyosteoarthritis, Morbus Dupuytren, carpal tunnel syndrome, or stenosing tenovaginitis. Other baseline characteristics of the patients included in this study are displayed in Table 1. We did not find any significant differences in demographic variables between responders and nonresponders (Supplemental Table 2).

**Table 1.** Patient Characteristics.

Total amount of patients, n = 74	
*Variable*	
Age, mean (SD)	57 (8.4)
Sex, No. (%)	
Male	22 (30)
Female	52 (70)
Duration of symptoms, mo, median [IQR]	18 [10-48]
Contractual h, median [IQR]	28 [20-40]
Surgery on the dominant hand, No. (%)	
Yes	45 (61)
No	29 (39)
Diagnosis, No. (%)	
Primary degenerative	55 (74)
Post-traumatic	19 (26)
Type of implant, No. (%)	
Silicone	28 (38)
Surface replacement	46 (62)
Operated on, No. (%)	
One finger	66 (89)
More than 1 finger	8 (11)
Hand comorbidity, No. (%)	
Yes	41 (55)
No	33 (45)
Physical occupational intensity, No. (%)	
Light (eg, office work)	32 (43)
Medium (eg, working in a store)	33 (45)
Heavy (eg, construction work)	9 (12)

*Note.* IQR = interquartile range.

**Figure 1.**
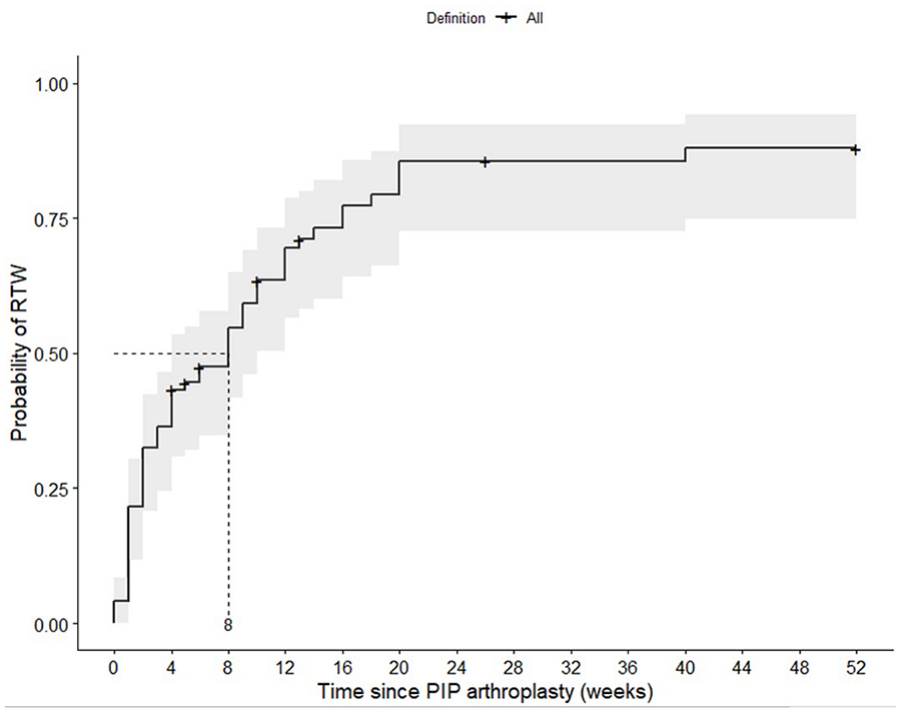
Kaplan-Meier plot including medians with 95% confidence intervals on the RTW after PIP joint arthroplasty in weeks. Half of the patients who underwent PIP arthroplasty returned to work within 8 weeks after surgery. The probability of returning to work within the first year after surgery was 88%. *Note.* RTW = return to work; PIP = proximal interphalangeal.

The median time to RTW for at least 50% of the original contractual hours for patients who underwent PIP joint arthroplasty was 8 weeks (interquartile range [IQR]: 4-10) (Figure 1). In the first year, the probability of RTW was 88%.

**Figure 2.**
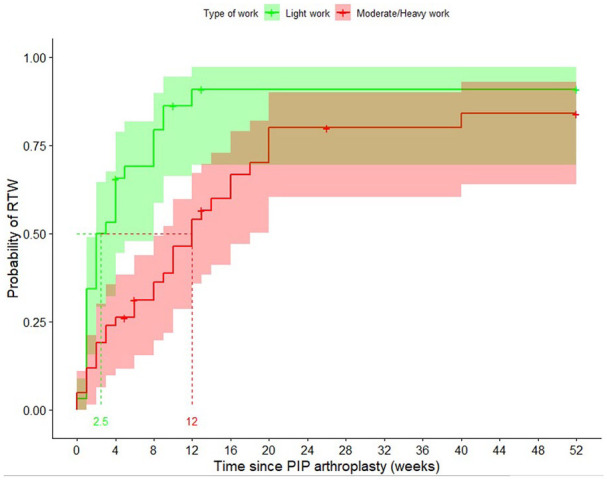
Univariable Kaplan-Meier plot including medians with 95% confidence intervals on the RTW after PIP arthroplasty based on physical occupational intensity. Patients with a light physical occupation returned to work after a median of 2.5 weeks (green line), and patients with a medium/heavy physical occupation returned to work after 12 weeks (red line). *Note.* RTW = return to work; PIP = proximal interphalangeal.

The median time until RTW for subgroups per factor is displayed in Table 2. In univariable analysis, the following variables significantly influenced the RTW: patients’ physical occupational status (light physical occupation, median: 2.5 weeks [IQR: 2-8] vs medium or heavy physical occupation, median: 12 weeks [IQR: 9-18] weeks, *P* < .001), sex (men, median: 2 weeks [IQR: 2.5-12] vs women, median: 9 weeks [IQR: 6-16], *P* = .020, Figure 2), and MHQ scores as displayed in Table 3. There was no significant difference in occupational status between men and women (*P* = .14). The factors such as age (*P* = .078), diagnosis (*P* = .9), the type of implant (*P* > .99), surgery on the dominant hand (*P* = .3), duration of symptoms (*P* = .65), and hand comorbidity (*P* = .7) did not influence the RTW in this group of patients.

**Table 2.** Median Time Until Return to Usual Work for Greater Than 50% of the Usual Contract Hours for Subgroups.

Variable	Median time to RTW (95% CI)	1-y cumulative RTW (%)
Age, y		
<57 (n = 33)	12 (6-20)	85
≥57 (n = 41)	4(2-8)	90
Sex		
Female (n = 52)	9 (6-16)	83
Male (n = 22)	2.5 (2-12)	100
Type of work		
Light (n = 32)	2.5 (2-8)	91
Moderate/heavy (n = 42)	12 (9-18)	84
Treatment side		
Dominant (n = 45)	6 (3-10)	89
Nondominant (n = 29)	9 (4-20)	90
Symptom duration, mo		
<18 (n = 34)	9 (3-16)	80
≥18 (n = 40)	6 (4-10)	95
Comorbidity		
No (n = 33)	10 (4-14)	93
Yes (n = 41)	6 (3-10)	86
Diagnosis		
Post-traumatic (n = 19)	9 (2-NA)	84
Primary degenerative (n = 55)	8 (4-10)	89
Implant		
Silicone (n = 28)	8 (3-18)	91
SR (n = 46)	7 (4-12)	84
MHQ total^a^		
<52 (n = 32)	12 (4—NA)	72
≥52 (n = 36)	4.5 (2-10)	100
MHQ hand function^a^		
<53 (n = 34)	5 (4-12)	87
≥53 (n = 34)	9.5 (3-18)	87
MHQ pain^b^		
<40 (n = 31)	9 (6-NA)	62
≥40 (n = 38)	4 (2-12)	100
MHQ work^b^		
<60 (n = 29)	12 (8-NA)	74
≥60 (n = 40)	4 (2-10)	100
MHQ ADL^a^		
<69 (n = 35)	8 (4-16)	81
≥69 (n = 33)	4 (2-12)	100
MHQ satisfaction^a^		
<33 (n = 31)	8 (4-16)	82
≥33 (n = 37)	6 (2-13)	92
MHQ aesthetics^a^		
<63 (n = 40)	10 (6-16)	93
≥63 (n = 28)	2.5 (2-10)	94

*Note.* Continuous variables were categorized based on the median value. RTW = return to work; CI = confidence interval; MHQ = Michigan Hand Outcomes Questionnaire; SR = surface replacement; NA = not applicable; ADL = activities of daily living.

a Missing in 6 patients.

b Missing in 5 patients.

**Table 3.** Univariable Cox Regression Analysis of Michigan Hand Outcomes Questionnaire Baseline Scores on Return to Work After Proximal Interphalangeal Joint Arthroplasty.

MHQ	Preoperative hazard ratio	95% confidence interval	*P* value
Total	1.024	1.006-1.043	.*009*
Hand function	0.996	0.984-1.015	.958
Pain	1.023	1.007-1.040	.*005*
Work	1.021	1.010-1.032	*<.001*
ADL	1.008	0.996-1.020	.205
Satisfaction	1.009	0.994-1.024	.266
Aesthetics	1.013	1.001-1.024	.*035*

*Note*. MHQ = Michigan Hand Outcomes Questionnaire; ADL = activities of daily living. Italic values are statistically significant (*P* < .05).

**Figure 3.**
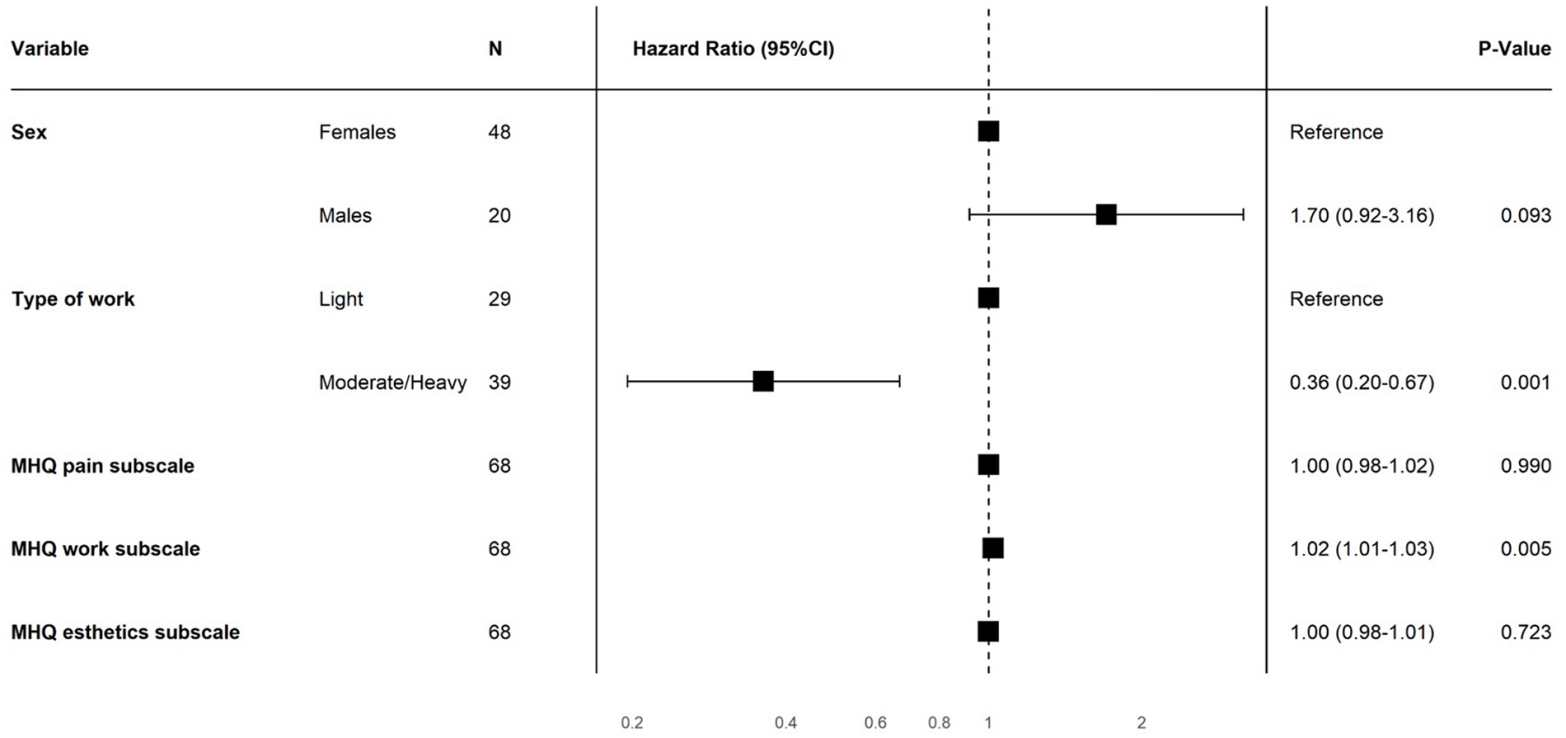
Multivariable Cox proportional hazards analysis. Physical occupational intensity (hazard ratio [HR]: 0.36, *P* = .001) and the baseline MHQ work scores (HR: 1.02, *P* = .005) were independently associated with return to work. *Note.* CI = confidence interval; MHQ = Michigan Hand Outcomes Questionnaire.

In multivariable Cox regression analysis, only physical occupational status (HR: 0.36, *P =* .001) and the baseline MHQ work score (HR: 1.02, *P* = .005) were associated with RTW (Figure 3), meaning that patients with a medium or heavy physical occupation returned to work later than patients with light physical occupation, and patients with better baseline MHQ work scores returned to work earlier than patients with worse baseline MHQ work scores. The chance of returning to work during the study period when a patient has a medium/heavy physical occupation is 36% smaller than a patient who has a light physical occupation.

## Discussion

Half of the patients who underwent PIP arthroplasty returned to work within 8 weeks after surgery. Patients with medium or heavy physical occupations returned to work later than patients with light physical occupations (HR: 0.36, *P* = .001). Besides, a higher MHQ work score at baseline led to an earlier RTW (HR: 1.02, *P* = .005). In this study, no other factors influenced the RTW.

In an earlier study, we found that the use of SR implants led to significantly higher patient-reported hand function scores but more swan neck deformities 1 year after surgery.^1^ In this study, the type of implant did not influence patients’ RTW, suggesting that the differences found for clinical outcomes did not affect RTW.

There were no previously published articles regarding RTW after PIP arthroplasty available for comparing our results. However, a previously published study has reported the RTW after trapeziectomy for thumb base osteoarthritis.^22^ They found that surgery on the nondominant hand, light physical occupational intensity, and higher MHQ work and hand function scores of the unoperated hand preoperatively led to an earlier RTW. Only the influence of patients’ physical occupational intensity on RTW is in line with the results of our study. Two other previously published studies assessed factors associated with RTW; however, they performed wrist surgery. They are less comparable to our study regarding pathophysiology and surgical procedure, but they did use the same method to assess factors associated with RTW and found that physical occupational intensity is an influencing factor. From this, we can conclude that it is essential for a surgeon, when treating patients with small joint osteoarthritis, to know the patient’s physical workload to inform a patient more adequately about the average time of recovery needed before returning to work.

We also found some differences when comparing our study with the previously published study of Van Der Oest et al.^22^ Their results showed an earlier RTW when patients were operated on the nondominant thumb. In our study, hand dominance was not associated with the RTW. A possible explanation might be that the thumb is essential for grip and pinch, and people rely on their dominant hand for daily activities and, in many cases, work. It is harder to relieve the thumb than other fingers in daily use, especially when the dominant hand is involved. Surgery on the dominant thumb may therefore lead to more disruption at work. The fact that 78% of patients undergoing trapeziectomy returned to work within 1 year after surgery versus 88% of patients in our study supports this theory.

In our study, besides the physical work intensity, only the pre-existent MHQ work score is of influence on the RTW, meaning that those patients who experienced fewer limitations in performing work preoperatively due to hand problems were able to resume normal work sooner than patients who experienced more problems during work preoperatively. It is crucial to assess the type of work and the ability to work preoperatively to better inform the patient about the probability of returning to the original work postoperatively.

Other factors than the ones studied in this cohort might also affect the RTW, such as the instructions of the patient’s surgeon, hand therapists, the demands of their employer, and possible worker’s compensation. In a previously published study among patients undergoing carpal tunnel release, the surgeons’ recommendations were the strongest predictors of a delayed RTW.^33^ Patients without workers’ compensation resumed work twice as fast as patients with workers’ compensation after internal fixation of the scaphoid.^34^ Future studies should also implement these factors when assessing the RTW after PIP arthroplasty.

Our study has some strengths and limitations. A strength of our study is that data were collected longitudinally on standard follow-up moments, and the influence of several factors on the RTW after PIP joint arthroplasty was assessed. Besides this, the survival analyses have the advantage of dealing with loss to follow-up and minimizing bias. Patients who reached retirement or did not complete additional questionnaires were censored but were still included in this study for the period that they provided data. Also, to detect possible confounders, we performed a multivariable analysis. One of the limitations is that we estimated the RTW with subjective questionnaires, for no information from public services was accessible. Besides, only 44% of patients who underwent PIP arthroplasty had a paid occupation before surgery and were therefore eligible for inclusion, leading to a limited study population. Besides this, due to the study’s design in which we aim not to burden patients with visiting the clinic, online questionnaires were sent, and 28 patients did not complete the RTW questionnaire. We compared responders with nonresponders and found no differences between the 2 groups, minimizing the risk of selection bias. In addition, we could speak of a limitation in terms of the surgeon’s recommendations regarding sick leave. In our country, the surgeon and hand therapists give advice and instructions on movement and weight bearing, but the independent occupational physicians advise patients on returning to work. These different opinions are mostly unverifiable and could also affect the RTW.

The information described in this study contributes to preoperative consultation and shared decision-making. Surgeons should keep in mind the patient’s occupational intensity and preoperative patient-reported work score when informing about the estimated time until they can RTW. Future studies should focus on assessing factors influencing the RTW after PIP arthroplasty in a larger cohort.

## Supplemental Material

sj-docx-1-han-10.1177_15589447221141485 – Supplemental material for Type of Work and Preoperative Ability to Perform Work Affect Return to Usual Work Following Proximal Interphalangeal Joint Arthroplasty for Osteoarthritis Supplemental material, sj-docx-1-han-10.1177_15589447221141485 for Type of Work and Preoperative Ability to Perform Work Affect Return to Usual Work Following Proximal Interphalangeal Joint Arthroplasty for Osteoarthritis by Bo J. W. Notermans, Joris S. Teunissen, Ruud W. Selles, Luitzen H. L. de Boer, the Hand Wrist Study Group and Brigitte E. P. A. van der Heijden in HAND

sj-docx-2-han-10.1177_15589447221141485 – Supplemental material for Type of Work and Preoperative Ability to Perform Work Affect Return to Usual Work Following Proximal Interphalangeal Joint Arthroplasty for Osteoarthritis Supplemental material, sj-docx-2-han-10.1177_15589447221141485 for Type of Work and Preoperative Ability to Perform Work Affect Return to Usual Work Following Proximal Interphalangeal Joint Arthroplasty for Osteoarthritis by Bo J. W. Notermans, Joris S. Teunissen, Ruud W. Selles, Luitzen H. L. de Boer, the Hand Wrist Study Group and Brigitte E. P. A. van der Heijden in HAND
