# Supplementary material for: Type of Work and Preoperative Ability to Perform Work Affect Return to Usual Work Following Proximal Interphalangeal Joint Arthroplasty for Osteoarthritis
Source: Hand (N Y). 2022 Dec 20;19(4):648–55. doi: 10.1177/15589447221141485 (PMC11141417; doi:10.1177/15589447221141485)
Supplement: sj-docx-1-han-10.1177_15589447221141485 – Supplemental material for Type of Work and Preoperative Ability to Perform Work Affect Return to Usual Work Following Proximal Interphalangeal Joint Arthroplasty for Osteoarthritis [file sj-docx-1-han-10.1177_15589447221141485.docx]

Supplementary Table 1. Rehabilitation protocol

| Day 0 | Surgery + application cast |
| --- | --- |
| Week 1-2 (day 1-14) |  |
| Day 3-5 | Removal of the cast wound inspection |
|  | Application of splint (day & night)  * *If hyperextension is present – 10-30° extension block splint* |
|  | Active extension/flexion E/F 0/45 without resistance  * *when a flexion contracture is present only passive extension* |
|  | DIP extension/flexion with PIP in 0° |
|  | If necessary, start swelling reducing measures |
| Day 10-14 | Wound inspection + removal of sutures |
|  | **Planning of hand therapy (1-3/week) – *continue until week 12*** |
| Week 3-4 (day 14-28) | If necessary, start scar treatment |
|  | PIP flexion up to 60° while maintaining extension 0° |
|  | Advice regarding Activities of Daily Living, work and recreation |
| Week 5-6 (day 28-42) | PIP flexion up to 75° while maintaining extension 0° |
|  | Advice regarding Activities of Daily Living, work and recreation |
| Week 7-12 (day 42-84) | PIP flexion up to 90° while maintaining extension 0° |
|  | Reduction of the splint during the day, continue wearing a splint at night |
|  | Build up strength, loading capacity, and functionality |
|  | Advice regarding Activities of Daily Living, work and recreation |
| 3 months | **A check-up with surgeon, X-ray if indicated** |
| Month 3-6 | Continue range of motion, strength, stability, loading capacity, functionality |
|  | Reduce splint and stop wearing |
|  | Hand therapy if indicated |
| Month 7-12 | Optimise function if necessary |
