# Supplementary material for: Type of Work and Preoperative Ability to Perform Work Affect Return to Usual Work Following Proximal Interphalangeal Joint Arthroplasty for Osteoarthritis
Source: Hand (N Y). 2022 Dec 20;19(4):648–55. doi: 10.1177/15589447221141485 (PMC11141417; doi:10.1177/15589447221141485)
Supplement: sj-docx-2-han-10.1177_15589447221141485 – Supplemental material for Type of Work and Preoperative Ability to Perform Work Affect Return to Usual Work Following Proximal Interphalangeal Joint Arthroplasty for Osteoarthritis [file sj-docx-2-han-10.1177_15589447221141485.docx]

| Supplementary Table 2. Responder analysis | | |  |  |  |  |
| --- | --- | --- | --- | --- | --- | --- |
| **Characteristics** | | **Non-responders** | | **Responders** | | **p-value** |
|  |  | *Not included* | | *Included* |  |  |
| *Number of patients* | | 28 |  | 74 |  |  |
| Age, mean (SD) | | 53 (12) |  | 57 (8.4) |  | 0.116 |
| Males, n (%) | | 6 (21) |  | 22 (30) |  | 0.555 |
| Duration of symptoms, median [IQR] | | 12 [9 - 33] |  | 18 [10 - 45] | | 0.316 |
| Surgery on non-dominant hand, n (%) | | 16 (57) |  | 29 (39) |  | 0.160 |
| Physical occupational intensity, n (%) | |  |  |  |  | 0.139 |
| Light (e.g. office work) | | 10 (36) |  | 32 (43) |  |  |
| Medium (e.g. working in a store) | | 10 (36) |  | 33 (45) |  |  |
| Heavy (e.g. construction work) | | 8 (29) |  | 9 (12) |  |  |
